# Computational cloning of drug target genes of a parasitic nematode, *Oesophagostomum dentatum*

**DOI:** 10.1186/1471-2156-14-55

**Published:** 2013-06-18

**Authors:** Nathan M Romine, Richard J Martin, Jeffrey K Beetham

**Affiliations:** 1 Departments of Veterinary Pathology, College of Veterinary Medicine, Iowa State University, Ames, IA 50011, USA; 2 Departments of Biomedical Sciences, College of Veterinary Medicine, Iowa State University, Ames, IA 50011, USA

**Keywords:** Transcriptome, *In silico* sequence, Nematode, Anthelminthic, Drug resistance

## Abstract

**Background:**

Gene identification and sequence determination are critical requirements for many biological, genomic, and bioinformatic studies. With the advent of next generation sequencing (NGS) technologies, such determinations are predominantly accomplished *in silico* for organisms for which the genome is known or for which there exists substantial gene sequence information. Without detailed genomic/gene information, *in silico* sequence determination is not straightforward, and full coding sequence determination typically involves time- and labor-intensive PCR-based amplification and cloning methods.

**Results:**

An improved method was developed with which to determine full length gene coding sequences *in silico* using *de novo* assembly of RNA-Seq data. The scheme improves upon initial contigs through contig-to-gene identification by BLAST nearest–neighbor comparison, and through single-contig refinement by iterative-binning and -assembly of reads. Application of the iterative method produced the gene identification and full coding sequence for 9 of 12 genes and improved the sequence of 3 of the 12 genes targeted by benzimidazole, macrocyclic lactone, and nicotinic agonist classes of anthelminthic drugs in the swine nodular parasite *Oesophagostomum dentatum*. The approach improved upon the initial optimized assembly with Velvet that only identified full coding sequences for 2 genes.

**Conclusions:**

Our reiterative methodology represents a simplified pipeline with which to determine longer gene sequences *in silico* from next generation sequence data for any nematode for which detailed genetic/gene information is lacking. The method significantly improved upon an initial Velvet assembly of RNA-Seq data that yielded only 2 full length sequences. The identified coding sequences for the 11 target genes enables further future examinations including: (i) the use of recombinant target protein in functional assays seeking a better understanding of the mechanism of drug resistance, and (ii) seeking comparative genomic and transcriptomic assessments between parasite isolates that exhibit varied drug sensitivities.

## Background

Helminth infection of the gut of humans and domestic animals is a global concern with tremendous social and economic costs [1]. Treatment of either humans or animals may involve administering anthelminthic drugs, typically from 1 of 3 main drug classes: the benzimidazoles (which selectively bind nematode beta-tubulin), macrocyclic lactones (which allosterically activate glutamate-gated chloride channels present in nematodes), or nicotinic agonists (which selectively activate subtypes of nematode nicotinic acetylcholine receptors; nAChRs) [2,3]. Not yet in widespread use are two more recently developed drugs monepantel [4] and derquantel [5] that represent amino-acetonitrile and spiroindole drug classes, respectively; both of these compounds have sites of action on subtypes of nematode nicotinic receptors other than the nematode levamisole receptor subtype [3] and have been promoted as ‘resistance-busting’. Of particular concern is that resistance has developed to members of each of the typically used drug classes [6,7]. Studies of anthelminthic drugs, many performed in the non-parasitic nematode *Caenorhabditis elegans*, have identified a number of genes/proteins that are drug targets of benzimidazoles (*ben-1*), macrocyclic lactones (*avr-14, avr-15, glc-1, glc-2, glc-3, glc-4*), and the nicotinic agonist levamisole (*lev-1, lev-8, unc-29, unc-38, unc-63*) (reviewed in [7,8]). From studies like these it appears that the molecular basis of susceptibility to macrocyclic lactones and levamisole is polygenic.

Although studies in *C. elegans* have provided important insights about drug-targets and drug-sensitivity, they are free-living bacteriovores and not parasitic worms. Therefore, there is great interest in extending drug sensitivity studies to the parasitic nematodes. One major hindrance to such efforts has been the lack of genome/gene information that is available for the parasitic nematodes. Consequently, studies requiring sequence determination typically include the time- and labor-intensive steps of: (i) identifying the target gene from among known genes of genetically close organisms, (ii) aligning target gene sequences to determine regions of high similarity, (iii) designing DNA primers to those regions, and finally, (iv) amplifying the target using PCR and a single pair of primers, cloning and sequencing the cloned product.

A modification of this approach, one having the potential to reduce the time and expense required to identify multiple gene sequences, is to use RNA-Seq data to build the target nematode gene sequences *in silico*, i.e., by computational methods. The next-generation sequencing (NGS) technique of RNA-Seq economically produces large amounts of sequence data, albeit comprised of very short (50-150 base) reads. When applied to an organism having a known genome, RNA-Seq sequence data can be computationally analyzed using software packages such as the commonly used Velvet package [9] to produce entire gene sequences *in silico*. Unfortunately, when applied to organisms for which little genome information is known, the output is more typically comprised of short contigs and contigs of lower quality [10].

An example of a parasitic nematode lacking a described genome is *Oesophagostomum dentatum*, a soil-transmitted helminth (STH) with a classic fecal-oral transmission route that causes minor nodules in the large intestine of swine and that is used as a parasite model for STH [11,12]. This report describes a reiterative method that builds upon assembly using the existing Velvet analysis software to allow greatly improved *in silico* determination of gene sequences from *O. dentatum*. The method was applied towards a determination of the sequence of 12 *O. dentatum* genes that, predominantly in *C. elegans*, have been identified as targets of the major classes of anthelminthic drugs (benzimidazoles, macrocyclic lactones, and nicotinic agonists). Establishing these sequences in *O. dentatum* facilitates downstream physiological and molecular studies.

## Results

### Generation of mRNA-seq read libraries

Separate mRNA-seq libraries were constructed from 5 μg of high quality total RNA (RNA Integrity Number ≥ 7.3) isolated from 58 adult male and 141 adult female worms. Given the absence of a sequenced genome of *O. dentatum* for use as a scaffold during subsequent assembly and analysis, paired-end sequencing was performed to facilitate contig-building steps. Similar numbers of 75-cycle paired reads were obtained for both libraries, (2.144 × 10^7^ for male, 2.159 × 10^7^ for female) for a combined total of more than 40 million reads. The lack of information about the exome size and complexity of *O. dentatum*, and that the RNA-seq libraries were not normalized, preclude estimation of the depth of coverage of the libraries; however, in broad approximation, the 3,100 × 10^6^ DNA base calls within the 40 × 10^6^ reads represent considerable coverage of mRNAs transcribed from the estimated 53-59 MB genome [13]. Reads were trimmed of read tags (added during library building to allow multiplexing during the sequencing run) and deposited at the NCBI sequence read archive: male library [GenBank:SRR393668] and female library [GenBank:SRR393669].

### Velvet assembly

**Figure 1:**
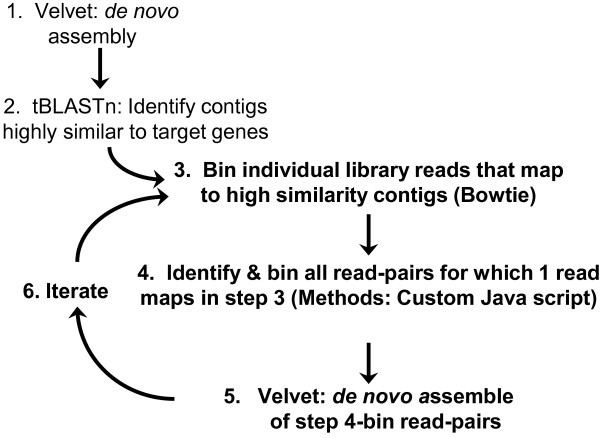
**Determination of gene sequences *in silico.*** (**1**) Individual data sets were assembled into contigs using Velvet. (**2**) BLAST searches for genes of the nAChR-pathway were carried out with a high cutoff (expect value = 1E^-10^) to identify contigs highly similar to the target genes. (**3**) Reads were individually mapped (using Bowtie) to high similarity contigs. (**4**) All paired-reads for which at least one read mapped to a contig (in Step- 3) were identified and binned using a custom Java program. (**5**) *De novo* assembly of Step- 4 sequences was performed using Velvet. (**6**) Iteration of Steps 3-5 was performed until the iteration resulted in no additional reads being mapped to the contig of interest.

The NGS assembler Velvet executed in the paired-end mode was used to assemble the 40 million reads from the combined male and female libraries into contigs (Figure 1, step 1). Combining the 2 libraries increased the potential number of reads that were identified for each target sequence while maintaining compliance with NCBI guidelines requiring submitted cDNA sequences be derived from a single strain. The paired-end mode was used for optimal *de novo* assembly, given the lack of a reference genome. Hashing, the early Velvet step in which individual reads are indexed into overlapping sequences (k-mers) of some length “k”, has significant impact on the length and quality of the contigs built during an assembly, with longer k-mers generally corresponding to greater specificity (i.e., that a detected alignment is actually correct) but lower sensitivity to detect alignments [9]. Because optimal k-mer length cannot be known a priori, multiple assemblies were performed using k-mers ranging from length 17 to 49.

The 12 genes of *C. elegans* shown to produce anthelminthic targets for which the homologous genes and sequences of *O. dentatum* were sought are shown in Table 1; the drug classes for which they are targets are shown in Table 2. To identify contigs exhibiting high similarity to the *C. elegans* target sequences separate BLAST databases, each comprised of the set of contigs produced by a single k-mer assembly, were built and then queried each database using tblastn and the protein sequences of the *C. elegans* target genes (Figure 1, step 2). A stringent “E-value” threshold of 1 × 10^-10^ was used within the tblastn search to limit false positive identifications; the E-value corresponds to the number of times a match of the same quality would be found by chance within the database. A custom java-script (Additional file 1) was used to collect those contigs whose corresponding high scoring pair (HSP) E-values were ≤ 1 × 10^-10^ and, for further stringency, to retain only those contigs whose region of alignment with the target protein exhibited ≥ 60% identity across ≥ 50% of the contig length. Some contigs that passed these filters exhibited high similarity to more than one target gene (data not shown), as would be expected given that some of the target genes are paralogous to one another. To unambiguously assign the filtered contigs to single target genes, contigs were queried (using blastx) against the *C. elegans* genome database and then gene identity was assigned based upon the match exhibiting the lowest E-value.

**Table 1:** Drug-target genes and high similarity initial-assembly contigs at multiple k-mers

***C. elegans *Target gene**				**21-mer HSPs**			**23-mer HSPs**			**25-mer HSPs**			**27-mer HSPs**		
**ID**	**Acc #**		**CDS len**	**#**	$\bar{X}$	**R**	**#**	$\bar{X}$	**R**	**#**	$$\bar{X}$$	**R**	**#**	$\bar{X}$	**R**
lev-1	CAB03148		1419	3	148	99-219	3	238	174-294	5	238	96-525	4	237	114-381
lev-8	CAB01685		1596	2	147	126-168	1	255	**255**	1	108	108		nd	nd
unc-29	CAB02308		1482	12	144	105-168	7	259	120-**480**	8	248	132-426	11	202	96-402
unc-38	CCD69819		1524	6	180	111-294	9	184	90-375	9	283	84-1098	8	339	87-1380
unc-63	CCD66192		1524	3	272	105-**576**	5	247	96-510	5	263	105-510	5	235	105-510
avr-14	CCD61323		1251	1	90	90	2	112	102-123	2	114	105-123	4	111	105-123
avr-15	CAB03329		1437	4	163	123-210	7	154	123-192	6	140	105-210	3	185	123-240
ben-1	CAB00853		1335	2	163	108-219	1	111	111	1	114	114	3	102	90-117
glc-1	CAB07361		1305	1	123	123	1	108	108	1	318	**318**	1	237	237
glc-2	CCD62432		1305	4	198	147-318	1	879	879	1	939	**939**	3	375	207-582
glc-3	CCD69051		1455	3	146	87-207	2	115	111-120	2	102	93-111	4	117	99-144
glc-4	CCD65896		1503	6	192	111-279	2	702	249-1155	2	702	249-1155	2	702	249-1155
	**29-mer HSPs**			**31-mer HSPs**			**33-mer HSPs**			**35-mer HSPs**			**37-mer HSPs**		
**ID**	**#**	$\bar{X}$	**R**	**#**	$\bar{X}$	**R**	**#**	$\bar{X}$	**R**	**#**	$\bar{X}$	**R**	**#**	$\bar{X}$	**R**
lev-1	5	219	93-420	11	150	99-330	9	181	114-255	8	207	102-558	6	334	138-657
lev-8			nd			nd			nd			nd			nd
unc-29	8	223	120-465	9	171	96-378	8	180	96-396	2	172	162-183	4	173	144-207
unc-38	7	493	90-**1473**	7	416	84-**1473**	6	472	108-**1473**	4	708	261-**1473**	3	890	360-**1473**
unc-63	3	227	105-297	4	285	105-414	3	275	105-414	1	414	414	2	277	141-414
avr-14	3	123	111-135	5	145	111-207	6	169	123-270	5	202	123-321	5	240	123-327
avr-15	5	156	90-243	7	153	96-**291**	4	132	93-213	4	138	96-243	4	108	108-111
ben-1	2	97	93-102	4	99	90-120	3	99	96-102	3	109	87-135	5	109	93-129
glc-1	1	237	237			nd	2	141	141-141			nd			nd
glc-2	3	261	147-393	2	439	135-744	2	475	207-744	2	387	171-603	2	336	333-339
glc-3	3	112	111-114	7	169	93-330	7	120	90-168	5	145	117-213	6	153	111-189
glc-4	2	702	249-1155	2	702	249-1155	2	702	249-1155	2	702	249-1155	1	972	972
	**39-mer HSPs**			**41-mer HSPs**			**43-mer HSPs**			**45-mer HSPs**			**47-mer HSPs**		
**ID**	**#**	$\bar{X}$	**R**	**#**	$\bar{X}$	**R**	**#**	$\bar{X}$	**R**	**#**	$\bar{X}$	**R**	**#**	$\bar{X}$	**R**
lev-1	3	564	339-807	1	1392	1392	1	1401	**1401**	2	619	345-894	3	245	219-270
lev-8			nd			nd			nd			nd			nd
unc-29			nd			nd			nd			nd			nd
unc-38	1	1473	**1473**	1	1473	**1473**	2	868	264-**1473**	1	1473	**1473**			nd
unc-63			nd			nd			nd			nd			nd
avr-14	1	390	**390**	1	219	219			nd			nd			nd
avr-15	2	111	111-111				1	120	120	1	174	174	1	174	174
ben-1	7	100	75-120	10	138	90-234	16	115	84-234	25	114	87-348	15	160	90-**453**
glc-1			nd			nd			nd			nd			nd
glc-2			nd			nd			nd			nd			nd
glc-3	3	618	84-**1383**			nd	1	378	378			nd			nd
glc-4	1	1428	**1428**	1	1428	**1428**	1	972	972	1	972	972	1	1428	**1428**

*C. elegans* target genes (name (ID), GenBank accession number (Acc #) and coding sequence length (CDS len)) used to BLASTx-query a database comprised of the initial *de novo* library assembly. For each k-mer (e.g. “21 mer HSPs”) are listed in columns the number of high scoring pairs identified (HSP; “#”), the mean HSP length in DNA bases (“$\bar{X}$”), and the range of HSP-lengths (“R”) with minimum and maximum length shown. Bold HSP-length values indicates the longest HSP identified among all k-mers for a given target gene. “nd” indicate no high-similarity HSPs were identified at that k-mer.

**Table 2:** Target gene identification and comparison

**Gene ID**	**Max HSP length (DNA)**		**Max Contig length (DNA)**		**Max Contig length (AA)**		**Length (AA)**	**Final vs *C. elegans *protein**		***O. dentatum***
	**Initial**	**Final**	**Initial**	**Final**	**Initial**	**Final**	***C. elegans***	**% ID**	**% full length**	**Acc #**
Levamisole target genes (nAChR subunits)										
lev-1	1401	1401	1580	1612	467	477	472	91	100	GACS01000001
lev-8	255	255	269	269	85	89	531	85	17	GACS01000002
unc-29	480	576	482	614	160	204	493	88	41	GACS01000003
unc-38	1473	1455	1607	1631	491	507	511	72	100	GACS01000004
unc-63	576	1248	578	1770	192	417	502	82	82	GACS01000005
Macrocyclic lactone, benzimidazole target genes										
avr-14	390	1299	629	1485	130	464	416	52	100	GACS01000006
avr-15	291	1242	560	1531	97	447	478	44	100	GACS01000007
ben-1_1	453	1308	453	1436	151	448	444	94	100	GACS01000008
ben-1_2	453	1317	453	1751	151	448	444	95	100	GACS01000009
glc-2	939	1215	994	1389	313	424	434	72	100	GACS01000010
glc-3	1383	1383	1724	2048	461	531	484	58	100	GACS01000011
glc-4	1428	1521	1628	1628	476	508	500	77	100	GACS01000012

Best initial high scoring pair (HSP) and contig lengths correspond to the longest HSP from the initial read assemblies (see bold values from Table 1) and the contig from which that HSP derives. “% full length” represents the percent of the comparator *C. elegans* protein that is represented within the final contig. “% ID” represents the percent identity as determined by pairwise alignment.

For each gene of interest, Table 1 indicates *C. elegans* target gene information including coding sequence length, and summarizes the BLAST results profile for each k-mer assembly from k-value 21 to 47; profiles for k-values of 17, 19, and 49 were unremarkable and are not shown. For each k-mer evaluated and each target sequence, the table indicates the number of HSPs identified, their mean length, and their range from shortest to longest. A best/longest HSP was identified for each target contig (as is indicated by bold font for R value), and no contig yielded more than 1 HSP for a target gene (data not shown). Relative to the length of the target gene coding sequences, the length of the corresponding longest HSP was quite short for 9 genes, and was near to completely full length for 3 other sequences (*lev-1*, *unc-38*, and *glc-4*). Table 1 demonstrates the impact that k-mer can have on the quality of assembly; for a given length k-mer, the quality of the assembly varied greatly, with (i) some k-mers producing many but short HSPs, (ii) other k-mers yielding few, but longer HSPs, and (iii) no single k-mer yielding the longest HSP (contig) for all target genes. Also as shown in Table 1, for some targets only a single k-mer produced the best HSP (e.g. glc-3 at 39-mer), whereas for other targets a range of k-mers yielded equivalent best HSPs (e.g. unc-38 at k-mers 29-45).

### Iterative-binning and iterative-assembly to optimize sequence determination

To improve the overall quality of the contigs (i.e., increase contig lengths by extension and by gap filling) additional computational steps of assembly were developed (Figure 1, steps 3-6). In outline, this involved identifying and binning all RNA-Seq library read-pairs for which at least 1 read matched a single contig for each target gene, and then using Velvet to reassemble those binned reads into contigs; this process was repeated until the output contigs exhibited no relative improvement. In detail, high-identity contigs that were identified in the initial Velvet assembly (at k-mer length 31 for all gene targets excepting *lev-*8, *glc-1*, and *glc-*3,which used k-mer lengths of 23, 25, and 39 respectively) were used in Step 3 (Figure 1) to identify and bin all library reads that mapped to those contigs; this process utilized the mapping program Bowtie set to increase the sensitivity of read identification by using a low quality threshold value (of 150) and by running it in unpaired-read mode. The unpaired-read mapping mode allowed inclusion of those reads that did not contribute to the prior contig (Table 1) for reason their pair read failed to map to that prior contig. Consequently, the reads binned in Step 3 included the paired reads along with a number of single reads for which their pair did not map; a custom Java-script collected into a single bin those reads that mapped as well as reads that did not map but whose read-pair did map (Figure 1, Step 4: Additional file 2). The Velvet program was then used to assemble contigs from the collected paired-end reads combined from both libraries (Figure 1, Step 5) using multiple length k-mers and coverage cutoffs to identify assembly conditions that produced a maximum contig length; this step involved minimal computational time given the small number of reads (< 2 * 10^5^) present among all bins. Bowtie mapping, read set collection and assembly were reiterated (Figure 1, Step 6) until a maximum contig length and maximum coverage (relative to the target genes) were achieved.

The dramatic effectiveness of the reiterative method is evidenced by comparison of contigs and corresponding best-HSPs of the final (iterative-derived) contigs to the initial contigs and to the *C. elegans* target sequence, as is shown in Table 2. Whereas the initial Velvet assembly yielded 3 sequences corresponding to nearly 100% of the target gene coding sequences (*lev-1, unc-38*, *glc-4*), reiteration yielded the full coding sequence of an additional 7 target sequences including a second isotype of *ben-1*, and yielded significant improvement to all other sequences excepting that of *lev-8*.

Interestingly, whereas the initial assembly yielded contigs that best-mapped by BLAST analysis to *glc-1* (Table 1), reiteration yielded an extended contig unambiguously identified by BLAST as the paralogous target gene *avr-15*. A closer examination of the initial contigs that were identified as *glc-1* revealed that their region of similarity to *glc-*1 was quite small and exhibited almost-as-high BLAST similarity to the paralog *avr-15*. Consequently, reads within the libraries provide evidence for transcripts for 2 *avr-15* variants and no *glc-1*.

The quality/accuracy of the *in silico* derived sequences can be inferred from the shared identity to target genes of *C. elegans* (Table 2, column “% ID”). Additionally, a direct indication of *in silico* sequence quality for 2 of the target genes (*unc-38* and -*63*) is possible because their sequence in *O. dentatum* had previously been determined (from PCR-amplicons). BLAST nucleotide comparison of the previously determined *O. dentatum unc-63* mRNA sequence [GenBank:HQ162136] to the corresponding 1770 nucleotide *in silico* sequence (Table 2, GACS01000005) identified a single alignment comprised of 1768 bases (including 30 non-identical bases) and no gaps. Of the non-identical bases, 20 were located within the coding sequence but only 1 of the 20 resulted in a change in the deduced protein sequence, a G_1411_A/ R_433_Q (numbering relative to HQ162136 DNA and corresponding protein sequences). Within the RNA-seq library reads this base call was invariant, i.e. of the 5 reads that mapped over the G_1411_A position, all were unique and all contained the “A” base call.

A similar comparison of the 1681 base *O. dentatum unc-38* mRNA sequence [GenBank:GU256648.1] to the corresponding 1631 nucleotide *in silico* sequence (Table 2, GACS01000004) identified a single alignment comprised of 1607 bases and no gaps, and which, when limiting the comparison to only the coding sequence, exhibited 3 base changes (i.e., 1521 of the 1524 coding sequence bases were identical). All 3 base changes result in amino acid changes: A_179_G/ Y_35_C, T_434_C/ Y_120_H, and T_1103_C/ F_343_S (numbering relative to GACS01000004 DNA and corresponding protein sequences). The other differences determined by alignment of the full sequences were that the GACS01000004 3’ UTR was shorter by 43 bases, and that the first 25 bases of the GACS01000004 75 base 5’ UTR was not contained within the 82 base GU256648.1 5’ UTR (suggesting the *in silico* sequence may represent a 5’ splice variant). To validate these differences within the 5’ UTR and the coding sequence of GACS01000004, reverse transcription polymerase chain reaction (RT-PCR) using a 5’ SL1 splice leader forward primer (see Methods) and an *unc-38* specific internal reverse primer were used to amplify an approximately 1600 base RNA segment (spliced leaders represent a set of invariant nucleotide sequences that are post-transcriptionally added to the 5’ end of many nematode mRNAs). The sequence of individual PCR clones fully recapitulated the GACS01000004 5’ UTR sequence as well as the 3 base changes within the coding sequence.

Further evidence for the validity of the *in silico* derived sequences was shown by RT-PCR amplification of *ben-1* and *avr-15* from RNA template using gene specific primers designed from the *in silico* sequence (Table 2, GACS01000008 and GACS01000007, respectively). The sequences of 9 *ben-1* PCR clones compared against that of GACS01000008 showed a single 1349 base no-gap alignment with 1307 identities and 42 variant base calls of which 6 resulted in a change in the deduced protein sequence: G_165_A/ V_49_I, G_512_A/ M_164_I, A_646_G/ D209G, T_840_C/ S_274_P, G_996_A/ V_326_M, C_1111_T/ A_364_V (numbering relative to GACS01000008 DNA and corresponding protein sequences). Interestingly, at each of the 42 sites of base variance, the majority of clone sequences called for an identical base to that of the *in silico* GACS01000008 sequence. In addition, at 21 of the 42 sites of base variance only a single PCR clone contained the variant base call. Thus the 42 base variants likely represent single nucleotide polymorphisms that are present within the population of *ben-1* RNAs examined. In the equivalent analysis of *avr-*15, the sequences of 2 PCR clones compared against that of GACS01000007 showed a single 1363 base no-gap alignment with 1320 identities and 43 variant base calls of which 6 resulted in a change in the deduced protein sequence: A_1089_G/ K_345_R, C_1103_T/ H_350_Y, C_1173_T/ V_373_A, T_1185_C/ V_377_A, A_1284_G/ K_410_R, C_1292_G/ L_413_V (numbering relative to GACS01000007 DNA and corresponding protein sequences). Among the 2 PCR clones, one contained only the V_373_A deduced change and otherwise was identical in translation to the protein deduced from GACS01000007, and the other clone contained all 6 deduced amino acid changes.

## Discussion

The data shown in Results demonstrate the efficient and successful use of an iterative *de novo* assembly of RNA-Seq data to determine *in silico* the sequence of 12 *O. dentatum* anthelminthic drug target genes. Selection of these particular genes was based upon their general importance to a range of studies investigating helminth drug targets (reviewed in [7,8]). The iterative assembly produced full length coding sequences for 9 target genes, whereas the Velvet assembly yielded full length (or nearly full-length) sequences for only a 3-gene subset of those 9 genes. A major utility of this process is that, as a computational process, it is scalable and should fit well to a variety of gene-characterization situations in *O. dentatum* or in any other nematode lacking a known genome sequence.

Related computational processes have been described with utility for producing output other than full length coding sequences. In one method, remapping of reads by identity to contigs within an initial assembly, and then reassembling contigs from those remapped reads, improved transcriptome assembly [14]; the desired output from that work was production of a general gene ontology. In another method, genome assemblies were variably improved via gap closure achieved by mapping paired-end reads and collecting pairs for which only one of the ends aligned to a contig [15].

As shown in Table 1, a number of k-mer lengths were used for initial library assemblies, demonstrating the dramatic effect of k-mer length on contigs. That said, as noted in Results, k-mer length 31 was used to build the contigs used in all target-gene resampling excepting that for *lev-8* (for which k-mer 31 returned no contigs; see Table 1) and *glc-3* (for which k-mer 31 contigs failed to support generation of resampled contigs representing the full target sequence). This suggests there is little need to use the best or longest contig as input to the resampling process. As a further test of this concept we were able to successfully build *avr-15* from a single approximately 300 base initial contig. Thus, there seems to be a reduced need to conduct full library assemblies over a wide range of k-mers when attempting to derive *in silico* the sequences for a set of target genes; instead, one stops when a limited set of k-mers that have been run produce a quality contig for each target.

The reiterative approach presented here may have utility on a larger scale to facilitate transcriptome projects in nematodes and other organisms that lack genomic information but for which more complete gene-transcript sequences are desired. A dynamic programming approach will likely be required in such extended application to accommodate the conditional filters used during reiterative binning and assembly, and to accommodate a broader range of possible contig-to-target sequence similarity scores (i.e., blast E-values). We note that for a gene family represented by many members within a single organism there can arise ambiguities in contig identification, something that was seen in the present study for some initially assembled nAChR subunit contigs (data not shown); that such ambiguities were not observed for any reiteratively assembled contigs logically suggests that the longer the contig sequence-lengths, the less the chance that ambiguities of identity assignment will occur.

Of interest to nematologists is the identification of a *lev-8*-like sequence in *O. dentatum*, since it has also been found in *C. elegans*[16] but not in *H. contortus*[17], a Clade V nematode that is considered more closely related to *O. dentatum* than is *C. elegans*[18]. This identification is validated by its 85% amino acid identity (Table 2) and 94% similarity to *C. elegans lev-8*; by comparison, it exhibited only 67% identity and 82% similarity to the highest BLAST-identified matching gene/protein in *H. contortus*, *acr-8* [GenBank:ABV68891]. These data suggest a loss of *lev-8* in *H. contortus* but not *O. dentatum*. Because data reported here identified only a partial *in silico* sequence for *lev-8* (<300 bp), it is uncertain whether *O. dentatum* contains a full length (functional) *lev-8* or only a vestigial (partial) *lev-8* sequence; if the latter, then *O. dentatum* may be close behind *H. c*ontortus in losing the gene; if the former, then given that the *lev-8* nAChR subunit has been shown in *C. elegans* to confer sensitivity to levamisole [19], one might predict a difference in levamisole sensitivity as a function of the presence, or absence of *lev-8*. While differences in the nAChR properties and drug sensitivities of closely related nematode species have been observed, the genetic basis for these differences are unknown.

## Conclusions

The reiterative approach presented here was effective in determining *in silico* longer sequence reads for 11 genes of a 12-gene set of drug target genes of *O. dentatum*, a nematode for which exists very little genomic or gene information; an initial Velvet assembly that yielded 3 full/nearly-full length sequences can be improved by reiteration to yield full coding sequences for 9 (or 10, including ben-1 isotype 2) target genes and improved sequences for the remaining genes. The reiterative approach is expected to have general application for the *in silico* gene identification/sequencing of any nematode for which detailed genetic/gene information is lacking. The identification of full coding sequences for the target genes enables further examinations including studies like (i) seeking to reconstitute functional proteins/systems for assessment *in vitro* (similar to [17]), (ii) seeking comparative genomic and transcriptomic assessments between parasites isolates that exhibit varied drug sensitivities; such studies are ongoing in our labs and those of collaborators.

## Methods

### Parasites

Adult *O. dentatum* worms were harvested from a pig (as described [20]) previously infected with an isolate that exhibits low sensitivity to levamisole [12]. Worms were counted, and separated by sex, by microscopic examination. To remove foreign contaminants, the separated (male versus female) worms were washed at least 3x in a pH 7.5 maintenance solution consisting of (mM): NaCl (150), KCl (2.7), CaCl_2_ (2), MgCl_2_ (0.3), PIPES (10), NaOH (13), glucose (11), NaHCO_3_ (12), penicillin 0.06 g L^-1^, streptomycin 0.1 g L^-1^. Worms were settled by gravity before removing each wash, then blotted dry and transferred to a 1.5 ml microfuge tube, weighed by difference, and processed for extraction of RNA.

### RNA isolation

Parasite samples resuspended in 1.0 ml TRI reagent (Molecular Research Center, Cincinnati, Ohio) were ground by mortar and pestle under liquid nitrogen then brought to a total volume of 2 to 3 ml TRI reagent. Total RNA was extracted from the TRI reagent according to the manufacturer’s instructions, including an additional centrifugation step for clearing insoluble material. Extracted RNA was treated with DNase I (New England BioLabs, Ipswich, Massachusetts) (10 min at 37°C, 10 min at 75°C), then re-extracted with TRI reagent and resuspended in diethylpyrocarbonate-treated water. RNA concentration, purity, and quality (RNA Integrity Number) were assessed on a 2100 Bioanalyzer (Agilent Technologies, Santa Clara, California).

### mRNA-Seq

The building of indexed, non-normalized, paired-end mRNA-seq libraries, and subsequent 75-cycle pyrosequencing on an Illumina GAIIx platform, were performed as a service by the DNA Facility (Office of Biotechnology, Iowa State University) using 5 μg total RNA (per sample). Male and female libraries were duplexed in a single sequencing lane.

### Genomics and bioinformatics

#### Assembly

Velvet version 1.1.06 [9] was used for contig assembly.

#### Similarity searching

BLAST algorithms [21] were used to compare contigs with sequences available in public databases including the National Center for Biotechnology Information (NCBI) to identify homologues from other nematodes, i.e., sequences returning BLAST expect values ≤ 1E^-10^.

#### Read mapping

64 bit Bowtie [22] version 0.12.7 was used to map reads for contig building.

#### Pairwise comparison

The Needle algorithm [23] was used for pairwise comparison.

#### Custom codes

Java 1: Java-script code to read BLAST output and collect contigs that pass identity thresholds from a contig source file: available at Additional file 1. The length and identity of BLAST HSPs collected are adjustable parameters in the source code and should be adjusted for the degree of relatedness between the organisms compared.

Java-2: Java-script code that reads Bowtie alignment file and collects mapped and orphan reads from read source file: available at Additional file 2.

### RT-PCR

RT-PCR was performed using gene specific primers for *ben-1* (ben-1 1 F: aacatgcgtgagatcgtgc, ben-1 1R: ctactcttctggataagcctcct) and *avr-15* (avr-15 1 F: atgcatggtctcctaattgt, avr-15 1R: caaatgaagtcagtattcctcatct) using Platinum Taq DNA Polymerase High Fidelity (Invitrogen, Carlsbad, California). RT-PCR was performed for *unc-38* using gene specific and SL1 splice leader primers (SL1: ggtttaattacccaagtttgag, unc-38 1R: caaggaggaaaatttatttcgagag) using Taq DNA Polymerase with ThermoPol Buffer (New England BioLabs, Ipswich, Massachusetts). Sanger sequencing was performed as a service by the DNA Facility (Office of Biotechnology, Iowa State University).

## Competing interests

The authors declare that they have no competing interests.

## Authors’ contributions

NMR performed the sequence assembly, wrote the custom scripts, and contributed to drafting of the manuscript. JKB and RJM contributed to the experimental design, analysis, and drafting of the manuscript. All authors read and approved the final manuscript.

## Supplementary Material

Additional file 1 **Java-script code to read BLAST output and collect contigs that pass identity thresholds from a contig source file.** Description of data: Java-script code to read BLAST output. Click here for file

Additional file 2 **Java-script code that reads Bowtie alignment file and collects mapped and orphan reads from read source file.** Description of data: Java-script code to read BOWTIE alignment file. Click here for file
